# Accidents among adults in Germany – Results from the accident module of the Panel ‘Health in Germany’ 2024

**DOI:** 10.25646/14028

**Published:** 2026-05-20

**Authors:** Anke-Christine Saß, Ronny Kuhnert

**Affiliations:** Robert Koch Institute, Department of Epidemiology and Health Monitoring, Berlin, Germany

**Keywords:** Accidents, Accidental injuries, Accident mechanisms, Accident locations, Accident events, Falls, Domestic accidents, Synonym Accidents at home Traffic accidents, Leisure accidents, Occupational accidents, Medical care, Inpatient care, Accident-related incapacity to work, Adults, Germany

## Abstract

**Background:**

Accidents cause human suffering, high costs and are a significant cause of death. In 2024, 34,060 people died in accidents in Germany. Data on non-fatal accidents is provided by the Panel ‘Health in Germany’ 2024 conducted by the Robert Koch Institute.

**Methods:**

In the accident module of the study, 26,923 people aged 18 and over provided information about accident-related injuries in the last twelve months that required medical treatment. Detailed information was recorded about the last accident. Accident prevalence (95 % CI) and other results on accidents (proportions with 95 % CI) were calculated.

**Results:**

Overall, 9.5 % of women and 10.5 % of men report at least one accidental injury. The difference is significant. The majority of accidents occurred at home (36.0 %), followed by traffic, leisure and work. Accidents occur significantly more often at home and less often at work for women than for men. There are differences in workplace accidents among men depending on their level of education. The most common cause of accidents is falls (30.4 %). Significantly more accidents involving falls occur among women than among men. Almost one in five accident victims was treated as an inpatient in hospital (18.7 %).

**Conclusions:**

The results highlight the social relevance of accidental injuries. Initial analyses of the accident module identify key accident locations and affected groups. Further evaluations will reveal prevention potential in greater detail.

## 1. Introduction

Accidents are a significant health problem in Germany. In 2024, a total of 34,060 people died from accidental injuries [1]. Over 30,000 accidental deaths occurred at home and during leisure time. This figure has almost tripled in the last 25 years [1], particularly due to the increase in fatal falls. In addition, there were 3,000 deaths in 2024 as a result of traffic accidents and deaths due to accidents at work and accidents in schools and other educational institutions. The high significance of accidental injuries is also reflected in the burden of disease. According to the results of the burden of disease estimate for Germany, 9.4 % of all years of life lost (difference between age at death and statistical remaining life expectancy) in men are attributable to injuries, while the figure for women is 5.4 % [2].

However, complete information on the extent of non-fatal accidental injuries is not available. A comprehensive annual estimate was last published by the German Federal Institute for Occupational Safety and Health (Bundesanstalt für Arbeitsschutz und Arbeitsmedizin, BAuA) in 2015 [3]. However, certain aspects can be derived from other data sources: In 2022, 8.1 % of the annual days of incapacity for work among employed members of the AOK statutory health insurance were attributable to injuries and poisoning (International Classification of Diseases, 10th Revision (ICD-10): S00 – T98) [4]. In addition, the treatment of injuries accounted for 5.2 % of direct healthcare costs in 2023, corresponding to expenditure of over € 25 billion [5].

Detailed knowledge of accident statistics in Germany is required in order to develop targeted prevention measures, including information on risk groups, frequent accident locations and causes. However, meaningful and differentiated statistics are only available for selected areas. Fatal accidental injuries are recorded in the German cause of death statistics on the basis of the injuries (ICD-10: S00 – T98) and their external causes (ICD-10: V01 – Y98). Despite known problems with completing death certificates ([6, 7]), this allows accident-related deaths to be recorded relatively well.

In the case of non-fatal accidental injuries, systematic recording only exists for certain accident locations. Reportable accidents at work, at school and on the way to and from work are well documented [8–10]. Injuries from traffic accidents registered by the police are also recorded statistically [11]. However, the information on the type and consequences of injuries remains rudimentary in German road accident statistics, as only a distinction is made between slightly injured, seriously injured and killed [12]. Representative and detailed information on accidents at home and during leisure time in Germany was last collected about 25 years ago by the German Federal Institute for Occupational Safety and Health [13].

Further information on accidents is provided by data from the healthcare system. Hospital discharge statistics by diagnosis record inpatient treatment cases due to injuries with corresponding ICD codes (ICD-10: S00 – T98) [14]. In contrast to the German cause of death statistics, only the injury is coded; there is no additional coding of the external cause (ICD-10: V01 – Y98). This means that it is not possible to distinguish between unintentional (accidental) and intentional injuries, nor to analyse the accident mechanism (e.g. fall). Corresponding information is also missing from the data on outpatient care, which has only been available to a limited extent to date. More specific information is provided by the Injury Data Base (IDB) [15] and the TraumaRegister DGU^®^ of the German Society for Trauma Surgery (Deutsche Gesellschaft für Unfallchirurgie, DGU) [16]. Both data sources provide information on the circumstances of the accident and the intention of the injury, i.e. whether the injury was unintentional (an accident) or intentional (violence against oneself or violence by others). However, they only cover accidents that occur in specific areas.

**Key messages**

► Accidents cause human suffering, high costs and are a significant cause of death.
► 9.5 % of women and 10.5 % of men suffer at least one accidental injury requiring medical treatment within a twelve-month period (stat. sig. difference).
► The majority of accidents happen at home (36.0 %), with the proportion of domestic accidents increasing with age.
► The most common cause of accidents is falling (30.4 %). The proportion of falls in accidents is significantly higher among women than among men.
► The most common accidental injuries are dislocations, sprains, strains and torn ligaments (40.9 %), with a fracture diagnosed in 22.1 % of accidents.
► Almost one-fifth of all accident victims are treated as inpatients (18.7 %), with the average length of stay in hospital being 9.8 nights.

Due to the high prevalence of accidental injuries and their far-reaching consequences, detailed regular recording and reporting of non-fatal accidental injuries is of great importance. The surveys of the Robert Koch Institute (RKI) on accidents are an important addition to the available data sources. The European Health Interview Survey (EHIS) [17, 18], which is conducted every five to six years by the RKI for Germany and is mandatory throughout Europe, contains only one indicative question on accidental injuries in the past year. Accidents were recorded in more detail in the accident module of the study German Health Update (GEDA) in 2010 [19]. With the conversion of the RKI health monitoring to a panel in 2024, it became possible to reintegrate the accident module into a health survey.

This article provides an up-to-date overview of non-fatal accidents among adults in Germany. Using data from the accident module of the ‘Health in Germany’ panel 2024, accident prevalence is reported by age, gender and education (last twelve months). Based on detailed information about the last accident, frequent accident locations and accident mechanisms, the type of accidental injuries and their treatment, as well as accident-related incapacity to work are described for different subgroups of participants.

## 2. Methods

### 2.1 Study design and sample

The panel ‘Health in Germany’ was established in 2024 as part of a recruitment study. The sampling was based on a double-stratified random selection: 359 primary sampling units, known as sample points, were randomly drawn from all municipalities in Germany, taking into account the federal state and BIK municipality size class [20] (first selection stage). In the second selection stage, addresses were drawn for each sample point, stratified by age group, from the address registers of the respective residents’ registration offices. The selected individuals were invited to take part in a short survey and asked for their consent to participate in future surveys as part of the panel [21]. 29 % of those invited registered for the panel. A mixed-mode approach was used, which enabled both online participation (Computer-Assisted Web interview, CAWI) and written-postal participation (Paper-and-Pencil Interview, PAPI) [22].

For the first annual wave of 2024, the RKI panel comprised 46,863 registered participants aged 18 and over: 24,881 women, 21,856 men, and 126 people with a different gender identity. In 2024, they were invited to participate in health surveys at three points in time (sub-waves) at intervals of around two months (see Infobox RKI Panel ‘Health in Germany’ 2024). In total, there were four different questionnaire variants (A, B, C, D) on different topics [23]. Questionnaire D contained questions about accidents. In a predefined rotating procedure, the people registered on the panel received one of the four questionnaire variants in each of the three sub-waves. Additional in-depth questions on sociodemographic information were included at the end of the first sub-wave questionnaire. The questionnaires were provided in German. Participation was possible online and in writing by post, whereby the method realised in the recruitment study determined the survey method for the 2024 annual wave. Data collection began in May 2024 with the first sub-wave and was completed at the beginning of January 2025 with the third sub-wave. The response rate (proportion of participants in relation to the people registered in the panel) was between 75 % and 81 % in the individual sub-waves according to the standards of the American Association for Public Opinion Research (AAPOR [24]). A detailed description of the methodology and response rate (also stratified by age and gender) can be found elsewhere [25].

### 2.2 Weighting

In order to correct for distortions due to selective participation and deviations of the sample from the population structure as much as possible, a multi-stage sample weight was calculated [26]. The initial recruitment study took into account the sample design and adjusted for population figures as of December 31, 2023 and the 2021 Microcensus. The following factors are taken into account: age, gender, federal state, BIK municipality size class [20], education (Comparative Analysis of Social Mobility in Industrial Nations, CASMIN [27]) and household size (single-person vs. multi-person household). Additionally, dropout weights were calculated using data from the recruitment study to counteract any distortion caused by selective participation in repeated sub-waves. Finally, adjustment weighting was performed again. The weighting was calculated separately for each questionnaire variant; the weights are defined for ages 18 and above. A detailed methodological description can be found elsewhere [26].

**RKI-Panel ‘Health in Germany’ 2024**

**Data holder:** Robert Koch Institute

**Objectives:** To provide comprehensive data on the health status, health-related behaviour and health care of the population in Germany, with the future possibility of longitudinal comparisons and analysis of trends over time

**Study design:** Panel study with a mixed-mode approach (online and written-postal participation)

**Population:** German-speaking population aged 18 and over in private households with main residence in Germany

**Sample:** Probabilistic/representative sample of the Health in Germany panel infrastructure

**Participants in the 2024 annual wave:** A total of 41,376 of the persons registered in the panel took part in at least one of the three sub-waves in 2024.

Questionnaire A: 14,759 women, 12,374 men,
  66 persons with other gender identities
Questionnaire B: 14,828 women, 12,258 men,
  61 persons with other gender identities
Questionnaire C: 14,709 women, 12,329 men,
  64 persons with other gender identities
Questionnaire D: 14,872 women, 12,368 men,
  66 persons with other gender identities

**Data collection:**

1st sub-wave: 28.05.2024 – 05.08.2024
2nd sub-wave: 12.08.2024 – 14.10.2024
3rd sub-wave: 28.10.2024 – 06.01.2025

More information at www.rki.de/panel-en

### 2.3 Variables relating to accidents

To describe the accident occurrence, the RKI Panel 2024 recorded the accident prevalence in the twelve months prior to the survey. Detailed information was requested about the last accident that required medical treatment. Each accident victim was asked 20 specific questions about the location of the accident, the mechanism of the accident, the injuries sustained, the treatment of the injuries and, if applicable, any subsequent incapacity to work following the last accident. In addition to providing information on the proportion of the population with specific accident-related injuries, data on the most recent accident also allow the overall pattern of accidents to be described.

The key distinguishing feature for accidents is the location of the accident, i.e. where an accident occurs (work, home, leisure, traffic). Work accidents exclude commuting accidents and include a small proportion of accidents in educational institutions. Home accidents are defined as accidents in the home or in the immediate vicinity of the home, i.e. in the garden or garage. Leisure accidents exclude traffic and home accidents. Traffic accidents include all accidents on public roads, squares or streets and thus include traffic accidents on the way to or from work and during leisure time. Accidents involving pedestrians, where no vehicle is involved, are also classified as traffic accidents.

The following categories were specified for recording accident mechanisms:

► Falls on level ground (e.g. on black ice on the road)
► Fall from height (e.g. from a ladder)
► Contact with a person (e.g. colliding with a teammate while playing football)
► Contact with an object (e.g. bumping into a cupboard or being hit by a car)
► Stab or cut injury
► Burn or scald
► Trapping or crushing
► Injury caused by an animal
► Overuse of a body part (e.g. sports injury caused by twisting or lifting a heavy object).

In the following analyses, other accident mechanisms that are very rarely reported are summarised under ‘other’ (poisoning, foreign bodies in the eye, mouth or ear, breathing difficulties, other accident mechanisms).

Accidental injuries were classified as (1) bone fracture, (2) concussion, (3) dislocation, sprain, strain, torn ligament, (4) open wound, superficial injury, contusion, (5) internal injury, (6) burn or scald, (7) poisoning, (8) near-drowning, (9) other.

With regard to medical care after the accident, respondents were asked whether they had been treated as inpatients in hospital and, if so, for how many nights. They were also asked whether they had undergone physiotherapy or rehabilitation. In addition, the number of days on which respondents were on sick leave as a result of their accidental injury was recorded.

Age, gender and educational status may be related to the accident and are therefore taken into account in the analyses. To describe gender differences, the Panel ‘Health in Germany’ surveyed both the gender assigned at birth and gender identity (including open-ended response options). The analyses by gender include people who identify as female or male. Gender-diverse people who do not fit into these categories are not shown separately in the analyses by gender due to the small number of cases. Respondents’ educational data was classified according to the CASMIN classification as low, medium and high education [27].

### 2.4 Statistical methods

The v5 data set from the RKI Panel 2024 was used for all calculations; all analyses were carried out using the Survey package in R 4.4.1. Case numbers, percentages, arithmetic means and 95 % confidence intervals (95 % CI) were calculated. Population-related proportions are reported as 12-month prevalences and must be distinguished from statements relating to accidents landscape (proportion of accidents). Group differences were assessed for statistical significance using a chi-square test or a t-test for survey data. Differences were considered statistically significant at p < 0.05. Cases with missing values are excluded from the statistical analysis.

## 3. Results

### 3.1 Description of the sample

26,923 individuals provided usable data on the occurrence of an accident (Table 1). 2,660 individuals reported at least one injury requiring medical treatment due to an accident in the last twelve months. These individuals were then asked more detailed questions about the accident and the resulting health problems.

### 3.2 Prevalence of accidental injuries

**Figure 1:**
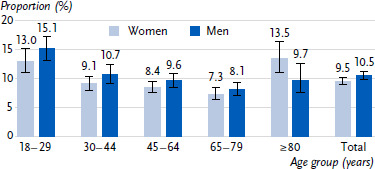
Persons with at least one accidental injury treated by a doctor in the last twelve months by gender and age (percentage with 95 % confidence intervals). Source: RKI Panel 2024, n = 14,650 women, n = 12,207 men

Approximately one in ten people (10.1 %) aged 18 and over suffer at least one accidental injury requiring medical treatment within a twelve-month period (Table 1, Figure 1). If extrapolated to the adult population in Germany [28], this would correspond to around 7 million people sustaining accidental injuries in 2024. Women (9.5 %) are slightly less likely than men (10.5 %) to be affected by non-fatal accidental injuries, but the difference is significant. The following applies to both women and men: younger adults under the age of 30 have significantly more accidents than older adults (aged 30 to 79). Young men between the ages of 18 and 29 in particular have a high accident risk, with a prevalence of 15.1 %. The risk of accidents decreases with age and increases again in the oldest age group, which is particularly evident among women. One in eight women aged 80 and over is affected (13.5 %). This is similar to the proportion in the youngest age group (13.0 % among women aged 18 to 29). Almost one third of accident victims have several accidents in twelve months (women 27.2 %, men 30.3 %). Looking at the education group to which the participants were assigned, there are no significant differences in the prevalence of accidental injuries.

### 3.3 Accident locations

**Figure 2:**
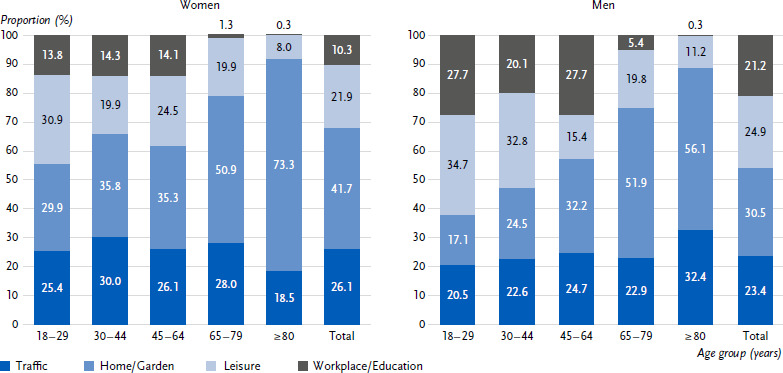
Accidents (last accident) by location among women and men in different age groups (proportion). Source: RKI Panel 2024, n = 1,361 women, n = 1,238 men

People who had had (at least) one accident were asked to provide more detailed information about their most recent accident. More than one in three accidents (36.0 %) happened at home, while a quarter were traffic accidents (24.6 %) and a quarter were leisure accidents (23.4 %). These were followed by accidents at work (16.0 %). From the age of 65 and especially from the age of 80, accidents occur significantly more frequently at home (Figure 2). This applies to both women and men.

There are clear gender differences in the locations of accidents. When an accident occurs, the workplace is significantly more likely to be the location of the accident for men (21.2 % vs. 10.3 % for women). For women, accidents are significantly more likely to occur at home or in the immediate vicinity of the home (41.7 % vs. 30.5 % for men). In the oldest age group (≥ 80 years), it can be observed that accidents involving men are more likely to occur in traffic. However, the total number of accident victims is low and the differences between the sexes are not significant.

### 3.4 Educational differences in accident rates

**Figure 3:**
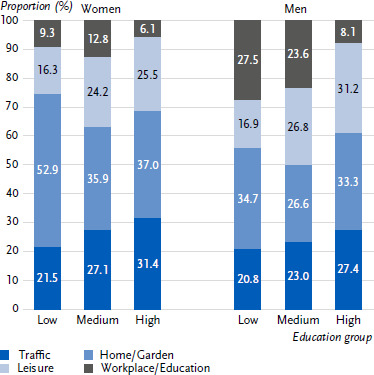
Accidents (last accident) by location among women and men in different education groups (CASMIN, proportion). Source: RKI Panel 2024, n = 1,360 women, n = 1,236 men (last accident) CASMIN = Comparative Analysis of Social Mobility in Industrial Nations

There are no differences in overall accident prevalence between women and men based on education. However, there are significant differences in terms of individual accident locations: a good quarter of accidents involving men in the low education group occurred at work (27.5 %) (Figure 3). For men in the high education group, the figure was only 8.1 %. The difference between these two education groups is significant. The opposite is true of accidents that occur during leisure activities. For men in the low education group, only one sixth (16.9 %) of accidents occurred during leisure time, compared with about one third (31.2 %) for men in the high education group; this difference is also significant. Among women, there are educational differences in domestic accidents. In the low education group, more than half of the accidents occurred at home (52.9 %), compared to only 35.9 % and 37.0 % in the medium and high education groups, respectively. The difference to the low education group is significant.

### 3.5 Accident mechanisms

Most accidents requiring medical treatment are due to falls (Table 2). Falls account for a significantly higher proportion of accidents among women than among men. 35.1 % of recent accidents among women were falls, while among men, around a quarter were falls (25.8 %). The risk of fall-related accidental injuries increases with age. Falls were reported significantly more often as the mechanism of the last accident in the 45-and-over age group than in younger age groups. In the 80-and-over age group, about half of all accidents requiring medical treatment are falls (women 52.7 %, men 48.9 %). Other important accident mechanisms in adults are overexertion (e.g. from lifting or carrying) and contact with objects (e.g. being hit by a ball). The accident mechanism of contact with objects is reported significantly more often by men than by women as the cause of their last accident.

### 3.6 Injuries in accidents

The most common accidental injuries are dislocations, sprains, strains or torn ligaments (40.9 % of recent accidents) and open wounds, superficial injuries or contusions (35.0 % of recent accidents) (Table 3). In 22.1 % of all accidents requiring subsequent medical treatment, a fracture is diagnosed. There are significant gender differences in the type of injury in the case of fractures (more common in women) and open wounds (more common in men). The spectrum of injuries changes with age. The risk of fracture in accidents increases, particularly in women. Approximately one third (36.9 %) of accidental injuries treated by a doctor in women aged 80 and over are fractures, compared with around one quarter (26.0 %) in men. Among the youngest age group (18 – 29 years), only around one in ten women and one in seven men reported a bone fracture resulting from their most recent accident (10.0 % vs. 14.5 %).

### 3.7 Medical care for accidental injuries

Accidents have significant medical and public health implications, not least due to the severity of the resulting injuries and the associated cost of care. Almost one in five accident victims was treated as an inpatient in hospital following their last accident (18.7 %). Among people aged 65 and over, around one third were admitted as inpatients following their last accident (30.1 % of 65- to 79-year-olds, 38.0 % of 80-year-olds and older). The difference compared to the youngest age group is significant (10.4 % of 18- to 29-year-olds). Gender-related differences in hospital use are not very pronounced. The average length of stay is 9.8 nights.

Around one third of accident victims (32.4 %) receive physiotherapy. One in six accident victims (15.8 %) receive medical rehabilitation (inpatient or day care/outpatient) following acute outpatient or inpatient care. No gender differences were observed. The use of physiotherapy services and inpatient rehabilitation measures due to accidents increases with age. The differences between the youngest and oldest age groups are significant.

### 3.8 Incapacity to work due to accidental injuries

Around 6.4 % of the working population are signed off work at least once a year due to an accident, for an average of 31.9 days. Accidents involving both women and men result in sick leave with a similar frequency. Two-thirds of working people who had an accident were subsequently on sick leave (women 64.5 %, men 64.4 %). There are no significant age differences, but there are differences between education groups. In the low education group, accidents lead to sick leave significantly more often than in the high education group (80.0 % vs. 45.1 % of accidents). On average, incapacity for work lasts longer in the low education group than in the high education group (36.5 days vs. 20.7 days, significant). These differences remain when differentiated by gender.

## 4. Discussion

### 4.1 Main results of the study

The RKI Panel 2024 analyses show that accidents and resulting injuries affect a large number of people, having a significant – and often underestimated – impact on healthcare provision and society as a whole. Approximately one in ten adults in Germany suffers an accidental injury requiring medical treatment within a twelve-month period: 9.5 % of women and 10.5 % of men. Younger men in particular are at high risk of accidents, with a prevalence of 15.1 %. The differences by gender and age are statistically significant. Among women, the prevalence of accidents shows a U-shaped curve with the highest incidence in the youngest and oldest age groups (13.0 % of 18- to 29-year-olds, 13.5 % of 80-year-olds and older). Our study asked detailed questions about the last accident. For both women and men, most accidents happen at home (women 41.7 %, men 30.5 %), with a significant difference between the sexes. This also applies to accidents at work: significantly more accidents happen at work for men than for women (women 10.3 %, men 21.2 %). The most common accident mechanism is a fall. Falls are significantly more common among women than men when they have accidents (35.1 % of women vs. 25.8 % of men). For people aged 80 and over, half of all accidental injuries requiring medical treatment result from falls, for both women and men. Overall, 22.1 % of accident victims report a fracture in their last accident. Almost one in five injured persons was treated as an inpatient in hospital (18.7 %). Patients with accidental injuries stayed in hospital for about one and a half weeks (average length of stay: 9.8 nights).

### 4.2 Strengths and limitations

One strength of this study is the high number of participants in the RKI Panel ‘Health in Germany’. Recruitment via the residents’ registration offices ensures a high degree of representativeness for the adult resident population living in Germany. A study-specific weighting factor is employed to correct for different probabilities of non-response. In addition to collecting data on accidental injuries (including key data on the circumstances of the accident), comprehensive sociodemographic information is also recorded, which can be linked in analyses. The data from the RKI Panel 2024 also reflects non-fatal accidents in the home and during leisure time, which are not included in any official statistics. This data source is unique in Germany.

Nevertheless, certain biases, such as selective non-participation (selection bias), cannot be completely ruled out. People who have suffered serious accidental injuries can only be included in the sample if they were already in a condition to participate in the survey during the survey period. Information on the consequences of accidents, such as the average length of stay in hospital, may therefore be underestimated. However, according to hospital discharge statistics by diagnosis, the length of stay in hospital due to injuries, poisoning and certain other consequences of external causes (ICD10: S00 – T98) was 7.0 days (all age groups) in 2023 [14] and thus below the values from the RKI panel 2024 (9.8 nights). Although the hospital discharge statistics by diagnosis also include children, who have shorter stays (2.0 days for 0- to 14-year-olds), this argues against distortions due to a potentially disproportionate proportion of slightly injured persons in our study. People in institutional living arrangements (e.g. nursing homes) are only accessible to a limited extent via registration register samples and are therefore probably underrepresented. Since the risk of accident-related injuries increases with age – as can be seen from the U-shaped prevalence curve for women in our evaluation – it is possible that the prevalence of accidental injuries is underestimated. In addition, people who are interested in health issues and those with more health-conscious behaviour are more likely to participate. Unregistered persons (e.g. homeless people) are not included in the sample from residents’ registration offices. Another limitation is that participation requires good written German skills.

A further limitation of health surveys arises from the restriction to self-reported data, which is influenced by the subjective perception and memory of the participants. Accidental injuries that occurred a long time ago or were less serious tend to be reported less frequently [29]. In order to exclude minor injuries, the present study only asked about accidental injuries that required medical treatment.

Due to the limited survey time, only detailed information on the last accident was recorded in the RKI Panel 2024. Our analysis of the overall accident situation is based on information from 2,660 most recent accidents. This represents a significant proportion of the total number of accidents, with 72.8 % of women and 69.7 % of men involved in accidents reporting that they had only been in one accident in the last twelve months.

### 4.3 Comparison with other surveys on accidents

It is difficult to compare the results of the RKI Panel with other data because there is no other source of data on all non-fatal accidents in Germany. The 2010 RKI survey, which used the same methodology, found a prevalence of 7.9 % of accidental injuries (requiring medical treatment) within twelve months [19, 30]. The Europe-wide EHIS (European Health Interview Survey) asks about non-fatal injuries at home, during leisure time and in traffic (with or without medical treatment). Accidents at work are not included. According to this survey, 12.7 % of all adults in Germany had an accidental injury in 2019 [31]. The prevalence of 10.1 % determined in our study seems plausible against this background, as it only includes accidents requiring treatment.

Data published by the European Statistical Office Eurostat, German cause of death statistics and other data sources show that younger people and men have an increased risk of fatal and non-fatal accidents [1, 19, 30–36]. In our study, men report accidental injuries in the last twelve months more frequently than women. This difference is significant, as was found in the last RKI survey [19, 30]. The highest accident prevalence was found among young men: 15.1 % of 18- to 29-year-olds had at least one accidental injury requiring medical attention within twelve months. However, with regard to accident-related deaths, Eurostat also reports that over 70 % of victims are older people aged 65 and over (2021: 71.4 %) [33]. Our data on non-fatal accidents shows that older people (especially women) are more affected in the current survey than in the last RKI survey from 2010 [19].

Most accidents occur at home. This result from our survey (36.0 % of recent accidents occurred at home) is consistent with the latest RKI survey from 2010 (30.2 % of accidents occurred at home, based on all accidental injuries in the last twelve months). Despite a different survey methodology, the Europe-wide EHIS survey also concludes that accidents at home and during leisure time dominate the accident statistics. In the 27 EU Member States (EU-27), 3.5 % of respondents reported a home accident, 4.3 % a leisure accident and 1.5 % a traffic accident within twelve months in 2019 [37]. The results of the surveys are consistent with the German cause-of-death statistics. According to these statistics, the vast majority of fatal accidents that occurred in 2024 happened at home or during leisure time (90.4 %) [1]. Traffic accidents account for 8.8 % of fatal accidents [1].

German road traffic accident statistics for 2024 document a total of 365,000 people with minor and serious injuries [38]. In our survey, 24.6 % of those involved in accidents reported that their last accident was a traffic accident. Extrapolated to the population, this means that at least 1.7 million people suffered injuries in traffic accidents. The difference can be explained by the fact that the German road accident statistics only include traffic accidents reported to the police [12]. In our study, we also record accidents that are not reported to the police, for example single-bicycle crashes. The RKI Panel is therefore more comprehensive and allows for further analysis, e.g. by type of traffic participation (on foot, by bicycle, by car, etc.) and the consequences of accidents not recorded by the police that resulted in injuries requiring medical treatment.

It is also difficult to compare our data with other surveys in the area of occupational accidents. In our study, 10.3 % of women and 21.2 % of men who had suffered an accident reported that their most recent accidental injury had occurred at work. The European EHIS survey excludes occupational accidents because most countries have special national recording systems. According to occupational accident statistics from the German Social Accident Insurance, around 740,000 reportable occupational accidents were recorded in the workplace in 2023. The higher incidence among men is also evident here: 72.6 % of those affected were men. In the case of fatal accidents in the workplace, 95.4 % of the victims were male [39]. Despite a long-term decline in non-fatal and fatal accidents at work, higher accident risks in high-risk labour market segments, such as the construction industry which has a significantly higher proportion of male employees, are consistently reflected in the statistics [10, 39]. Jobs in high-risk labour market segments often have a low status. In our study, the proportion of accidents at work among men in the low education group is significantly higher than among men with high education.

With regard to the accident mechanism, numerous surveys confirm the main finding of our study: falls are the most common cause of accidents. Both the 2010 survey conducted by the RKI [19, 30] and more recent studies and publications on fatal and non-fatal accidents cite falls as the most common accident mechanism [33, 39, 40–42]. According to the German cause of death statistics, falls are responsible for two-thirds of all accidental deaths. 21,176 fatal falls are reported for 2024 (ICD-10: W00 – W19) [1].

There is hardly any comparable data available on healthcare provision after an accident, which was also covered in our study. The 2019 EHIS found that 44.5 % of accident victims in Germany had been treated in hospital [43]. The data refers to the most serious accident in the last twelve months [44]. No distinction is made between whether the injured had to stay overnight in hospital or were only treated on an outpatient basis. In our study, 18.7 % of accident victims stated that they had been admitted to hospital following their most recent accident.

### 4.4 Implications for public health, prevention and policy

The results of the RKI Panel 2024 provide a comprehensive overview of accidents among adults in Germany and represent a key source of data for accident prevention. They provide relevant information for various stakeholder groups, including health policymakers, medical professionals, health and accident insurance companies, professional associations, urban and transport planners, sports organisations, and the industrial product safety sector. Basic data on accidents is available in the form of tables and graphs on the interactive web portal of the Federal Health Reporting (see Infobox 1). The analyses of the accident module illustrate that preventive measures must be adapted to age- and gender-specific patterns of accidents in different areas of life. In-depth analyses allow these differences to be characterised in detail and specific starting points for targeted prevention strategies to be identified.

Particular attention should be paid to accidents in the home and during leisure time. No population-based data has been available for these areas in Germany for several years. The last detailed representative survey by the Federal Institute for Occupational Safety and Health on accidents in the home and during leisure time was conducted in 2000 [13]. Ten years later, the accident module was included in the study GEDA 2010 conducted by the RKI [19]. The results now available and the planned further analyses thus close an important information gap and provide a basis for the development of evidence-based prevention measures. From a public health perspective, falls at home and during leisure time are particularly relevant in terms of frequency and severity (see also [19]). The Swiss Council for Accident Prevention (bfu), a foundation responsible for monitoring and preventing accidents outside the workplace, provides examples of suitable measures [40]. The website offers a wide range of preventive measures – information, flyers, campaigns for private households, institutions, authorities and accident prevention stakeholders. These include the guide ‘Making your home fall-proof. Safe living at any age’ [45]. Every two years, the bfu publishes a comprehensive report entitled ‘Statistics on non-occupational accidents and safety levels in Switzerland’, which contains current data [40]. Based on this, measures are designed for all age groups and accident locations, campaigns are launched and safety concepts are reviewed. Such an interlocking of data collection, evaluation, design of measures, communication, implementation and evaluation is not currently established in the field of home and leisure accidents in Germany. Availability of comprehensive, up-to-date data on the overall accident situation among adults is a first step in this direction.

### 4.5 Open questions and future research

There are plans to use the dataset for further in-depth analysis, particularly within the home and leisure sector. One topic that lends itself well to this is falls. Falls are the most common cause of accidents and affect all age groups, as Swiss data also shows. The proportion of falls in accidents increases with age [19, 40]. In addition to in-depth analyses of falls using data from the RKI Panel 2024, fatal accidents caused by falls are also a topic for future research. The number of deaths from accidents at home or during leisure activities is now almost three times higher than it was 25 years ago [1]. About two-thirds of these fatal accidents are caused by falls [46]. In Germany and many other European countries, there has been a significant increase in fatal falls in recent years [47, 48]. This cannot be explained solely by the ageing of the population. The extent to which changes in coding practices may also play a role remains to be investigated.

In addition, traffic accidents that are not recorded by the police are an important blind spot in accident statistics. Our data can help shed light on this. The RKI Panel 2024 asked whether the police were called if the accident took place on public roads, streets or squares. The mode of transport (e.g. on foot, by bicycle or by motor vehicle) was also recorded. Another focus will be on serious accidents (e.g. involving fractures or hospitalisation) and accidents involving employed persons.

In addition to the planned specific analyses, communicating the results to politicians and experts is a task of overriding importance. The perception of accidental injuries as a significant public health issue in Germany must be strengthened and the use of data for prevention and practice must be promoted. Accidents are preventable.

The vision of a world without serious accidents is formulated in the ‘Vision Zero’ concept. It has its origins in occupational safety and was transferred to the field of road safety in Sweden at the end of the 1990s [49]. In Germany, ‘Vision Zero’ has been incorporated into statutory occupational safety as a preventive strategy since the early 2000s. The concept also forms the basis of the road safety work carried out by the German Road Safety Council [49, 50]. Given that more than 30,000 people have died and millions more have been injured in accidents at home or during leisure activities in Germany, the Vision Zero initiative must be expanded. This requires systematic monitoring, including regular surveys with details of accident circumstances. Targeted campaigns, personalised advice and other specific preventive measures can help achieve Vision Zero’s goal of a world without serious accidents, where people are safer, healthier and more well-being.

**Infobox 1 Web portal of Federal Health Reporting**

The website www.gbe.rki.de/EN of Federal Health Reporting at the Robert Koch Institute (RKI) provides reliable information on the health situation of the population in Germany: timely, transparent and easily accessible. The focus is on noncommunicable diseases such as diabetes mellitus, cardiovascular diseases, cancer and mental disorders. It also presents factors that have an influence on health, such as health behaviour or social determinants. In addition, the web portal provides information on health care and contextual factors, such as food taxation or tobacco control measures, which also influence the health of the population.

The website currently includes over 60 indicators from health monitoring at the RKI and other data sources, which are interactively visualised and contextualised in short texts. The data is published as open data on GitHub and Zenodo. In addition, the website provides access to all RKI publications that are related to the topics on the website. The content is continuously expanded.

Further information on the topic of this article can be found on the web portal at www.gbe.rki.de/accidental-injuries

## Figures and Tables

**Table 1:** Sample of respondents^*^ (questionnaire D) and respondents with at least one accidental injury in the last twelve months – number of participants by gender, age and education group with 95 % confidence intervals. Source: RKI Panel 2024

	Respondents	Respondents with accidental injuries	Proportion with accidental injuries (95 % CI)
**Total**	**26,923**	**2,660**	**10.1 (9.6 – 10.6)**
**Gender**			
Women	14,650	1,384	9.5 (8.9 – 10.2)
Men	12,207	1,259	10.5 (9.8 – 11.3)
People with other gender identities	66		
**Age group**			
18 – 29 years	3,893	553	14.3 (12.8 – 15.9)
30 – 44 years	5,673	564	10.0 (9.0 – 11.1)
45 – 64 years	9,069	821	9.0 (8.2 – 9.9)
65 – 79 years	6,057	474	7.7 (6.9 – 8.5)
≥ 80 years	2,231	248	12.0 (10.2 – 14.1)
**Education group (CASMIN)**			
Low	5,093	444	9.3 (8.3 – 10.4)
Medium	12,891	1,292	10.5 (9.8 – 11.2)
High	8,888	921	10.4 (9.7 – 11.2)

CI = confidence interval, CASMIN = Comparative Analysis of Social Mobility in Industrial Nations

^*^who provided usable data on the occurrence of an accident

**Table 2:** Accidents (last accident) by accident mechanism in women and men (proportion with 95 % confidence intervals). Source: RKI Panel 2024, n = 1,345 women, n = 1,239 men

Accident mechanism (last accident)	Women		Men		Total	
	%	(95 % CI)	%	(95 % CI)	%	(95 % CI)
Fall (on level ground/from height)	35.1	(31.8 – 38.6)	25.8	(22.9 – 29.0)	30.4	(28.2 – 32.6)
Contact with object	9.8	(7.8 – 12.2)	15.3	(12.7 – 18.3)	12.5	(10.9 – 14.4)
Stab or cut wound	5.3	(3.9 – 7.1)	8.9	(7.0 – 11.3)	7.2	(5.9 – 8.6)
Overuse of a body part	17.2	(14.6 – 20.1)	17.2	(14.6 – 20.0)	17.0	(15.2 – 19.1)
Contact with person	3.2	(2.0 – 5.2)	4.3	(3.1 – 5.8)	3.8	(2.9 – 5.0)
Pinching or crushing	1.9	(1.1 – 3.2)	5.3	(3.6 – 7.9)	3.9	(2.8 – 5.4)
Injury caused by animal	6.5	(5.1 – 8.3)	3.3	(2.2 – 4.9)	4.9	(3.9 – 6.0)
Burn or scald	2.3	(1.3 – 3.9)	1.9	(1.0 – 3.7)	2.0	(1.4 – 3.1)
Other^*^	18.7	(15.5 – 22.5)	18.0	(15.2 – 21.1)	18.3	(16.1 – 20.7)

^*^Poisoning, suffocation/breathing difficulties, foreign bodies in the eye, mouth or ear and other accident mechanisms

CI = confidence interval

**Table 3:** Type of accidental injuries (last accident) in women and men (proportion with 95 % confidence intervals, multiple answers possible). Source: RKI Panel 2024, n = 1,374 women, n = 1,251 men

Accidental injury (last accident)	Women		Men		Total	
	%	(95 % CI)	%	(95 % CI)	%	(95 % CI)
Dislocation, sprain, strain, torn ligament	43.5	(40.2 – 46.8)	38.9	(35.5 – 42.3)	40.9	(38.6 – 43.4)
Open wound, superficial injury, contusion	30.3	(27.1 – 33.8)	38.9	(35.6 – 42.4)	35.0	(32.8 – 37.2)
Bone fracture	25.7	(22.9 – 28.7)	19.1	(16.3 – 22.2)	22.1	(20.3 – 24.0)
Internal injury	3.4	(2.4 – 5.0)	5.6	(4.2 – 7.5)	4.5	(3.6 – 5.6)
Concussion	4.0	(3.0 – 5.5)	4.9	(3.4 – 7.0)	4.5	(3.5 – 5.7)
Burn, scald	3.3	(2.2 – 5.0)	2.2	(1.3 – 3.9)	2.7	(2.0 – 3.8)
Poisoning	1.9	(1.2 – 3.0)	2.0	(1.2 – 3.2)	2.0	(1.5 – 2.8)
Near drowning	0.1	(0.0 – 0.6)	0.1	(0.0 – 0.8)	0.1	(0.0 – 0.4)
Other	12.2	(9.9 – 14.9)	14.0	(11.3 – 17.3)	13.3	(11 .5 – 15.4)

CI = confidence interval
